# Realization of tunable artificial synapse and memory based on amorphous oxide semiconductor transistor

**DOI:** 10.1038/s41598-017-04641-5

**Published:** 2017-09-08

**Authors:** Mingzhi Dai, Weiliang Wang, Pengjun Wang, Muhammad Zahir Iqbal, Nasim Annabi, Nasir Amin

**Affiliations:** 1 Ningbo Institute of Materials Technology and Engineering, Chinese Academy of Sciences, Ningbo, 315201 China; 2 Institute of Circuits and Systems, Ningbo University, Ningbo, 315211 China; 3 College of Physics and Electronic Information Engineering, Wenzhou University, Wenzhou, 325035 China; 4 GIK Institute of Engineering Sciences & Technology, Topi 23640, Khyber, Pakhtunkhwa Pakistan; 5 Department of Chemical Engineering, Northeastern University, Boston, USA; 6 Government College University Faisalabad, Faisalabad, Pakistan

## Abstract

Recently, advanced designs and materials emerge to study biologically inspired neuromorphic circuit, such as oxide semiconductor devices. The existence of mobile ions in the oxide semiconductors could be somewhat regarded to be similar with the case of the ions movements among the neurons and synapses in the brain. Most of the previous studies focus on the spike time, pulse number and material species: however, a quantitative modeling is still needed to study the voltage dependence of the relaxation process of synaptic devices. Here, the gate pulse stimulated currents of oxide semiconductor devices have been employed to mimic and investigate artificial synapses functions. The modeling for relaxation process of important synaptic behaviors, excitatory post-synaptic current (EPSC), has been updated as a stretched-exponential function with voltage factors in a more quantitative way. This quantitative modeling investigation of representative synaptic transmission bias impacts would help to better simulate, realize and thus control neuromorphic computing.

## Introduction

In recent years, a lot of advanced architectures have emerged as potential candidates for new generation of integrated circuits for various applications such as neuron computing, including three dimentional (3-D) integration^1–3^, quantum cellular automata^4–8^, defect tolerant architecture^9, 10^, molecular architecture and quantum computing^11–13^. However, they all have some limitations for use in the large scale circuits. For example, 3-D integration has difficulty to be test and measured. Quantum architectures have low temperature operation requirement and are sensitive to background noise. Defect tolerant architecture requires pre-computing tests. Molecular architecture has unclear mechanism and thus have limited the functionality to date. Complementary Metal Oxide Semiconductor ﻿(CMOS) transistors have been employed to simulate the representative synaptic transmission behaviors of synaptic devices such as excitatory post-synaptic current (EPSC)^14–21^. By modifying ionic fluxes in neurons and synapses, synaptic behaviors could be established in the human brain, such as signal processing, memory, and learning functions. A potential spike signal in a presynaptic neuron can trigger an ionic EPSC through a synapse, which may last for as much as milli-seconds in a postsynaptic neuron. The semiconductor materials devices with mobile ions can somewhat ﻿mimic the representative synaptic behaviors with fast increase signal and subsequently slower decade including EPSC, spike-timing-dependent plasticity (STDP), etc^15, 16, 21^. However, the appropriate control such as voltage dependence of representative synaptic transmission behaviors are rarely studied in details. Most of the plasticity behaviors studies generally focus on the spike time, spike pulse number, and neuron materials^16–21^.

Meanwhile, amorphous oxide semiconductor materials and transistors have a combination advantages of transparency, flexibility, a relatively fair mobility (>10 cm^2^/V · s) and room temperature processing with uniform properties in large scale production^22–26^. Therefore, these device structures are the promising candidate for electronics such as liquid crystal display and light-emitted-diode drivers. In this article, we propose amorphous In-Ga-Zn oxide (α-IGZO) transistors to simulate the synaptic devices^14–21^. Oxide semiconductor devices have been employed to simulate the synaptic plasticity behaviors and this mechanism could be understood as follows. Neurons and synapses are two basic computational units in the brain, whose plasticity transmissions depend on the existence of ions. For instanc﻿e, a synatic transmission could be regarded as initiating with the opening of voltage-gated calcium ion channels, where Ca^+2^﻿ ions diffuse inside the neuron. Then neurotransmitters were released ﻿from synaptic vesicles and diffuse through the synaptic gap. Subsequently, they bind to the receptor sites of Na^+^ gated ion channels at the post-synaptic neuron, which enable Na^+^ ions to diffuse inside the cell. Amorphous oxide semiconductor devices have metal ions components, which could be regarded to be similar with the case of neurons and synapses which also depends on the﻿ i﻿on transport. Therefore, the ions transport inside the amorphous oxide devices could be used to investigate the transmission of synapse and neurons signals^21, 22, 27^. EPSC is one of the common behaviors of the synaptic plasticity behaviors which has been  investigated^14–21^. Previous research studies have generally focused on stimula time, cycles or neuron categories. Leveraging these intriguing features, typical and tunable synaptic behaviors are investigated in our design, suggesting voltage dependency of these behaviors. This study could help better controlling the artificial synaptic devices, which are the fundamental components for neuromorphic computing systems.

**Figure 1:**
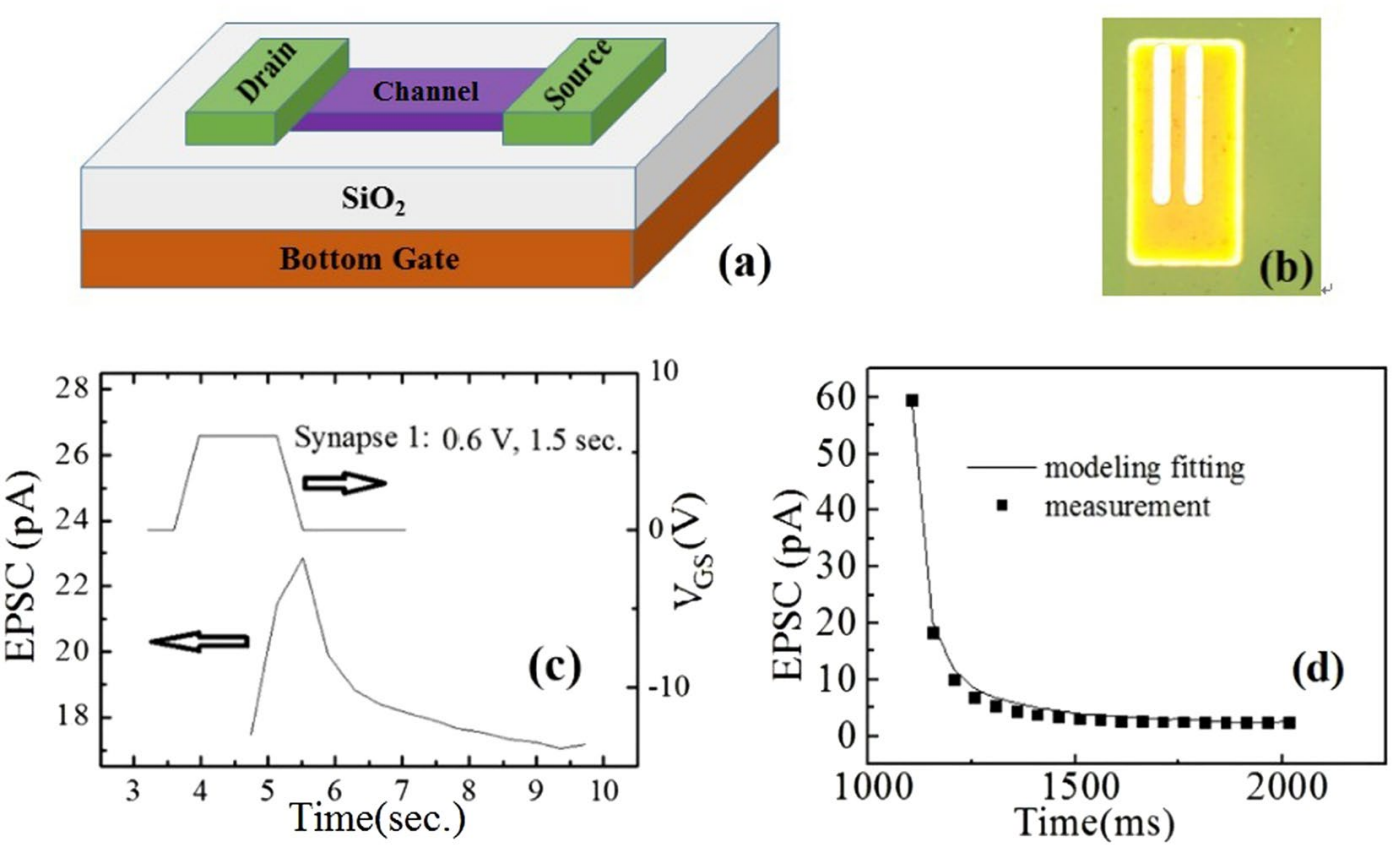
(**a**) The schematic of the 3D structure of the device with bottom gate, source, drain, and semiconductor channel, respectively. The conductive electrodes made of Ni/Au were used. The connection line between the drain and source is the semiconductor channel. (**b**) A representative image form the fabricated device with the bright white metal electrode as long as 800 μm. (**c**) EPSC triggered by presynaptic spike on bottom gate BG. (**d**) EPSC decay behavior with a fitting by stretched exponential function.

A 3D schematic idea of our artificial synaptic device is shown in Fig. 1a. The synaptic TFTs device based on In-Ga doped Zn-O (IGZO) was fabricated using AOS TFTs method^4^. Fig. 1b is a photo of the device which is actually fabricated. This typical representative EPSC records behavior in neuron rises immediately and falls relatively more gradually than when it rises in the time ranging from microseconds to miliseconds^15, 21^. Especially when the semiconductor has mobile ions, which shows similar trend as charging and discharging in a transistor or capacitor^14, 15, 21^. The EPSC is measured and investigated from the drain in our IGZO device after applying a trapezoidal voltage pulse at the bottom gate terminal as shown in Figure 1c. When the drain voltage is fixed, after a pulse applied on the gate terminal, the measured EPSC from the drain reaches a peal value and decreases gradually, which is similar to that in a biological excitatory synapse^14^. Here, IGZO is employed as the semiconductor channel of the TFTs. Therefore, the mechanism for the decaying EPSC could be attributed to the oxygen species diffusion/migration, which is suggested as the working mechanism for electrical properties shift of IGZO TFTs in previous publications^21, 22, 27^.

As shown in Fig. 1d, the decay of measured EPSC can generally fit well the line calculated by a stretched-exponential function^17^. This suggests that EPSC could be described as a stretched-exponential function. Here, we use EPSC as a representative synaptic transmission to study the voltage dependence of the synaptic plasticity relaxation behaviors. A standard stretched-exponential function is described as Eq. 1 ^17^.

1 $${\rm{I}}=({{\rm{I}}}_{0}-{{\rm{I}}}_{\infty })\exp [-{(\frac{{\rm{t}}-{{\rm{t}}}_{0}}{{\rm{\tau }}})}^{{\rm{\beta }}}]+{{\rm{I}}}_{\infty }$$

where τ is the retention time, t_0_ is the time when the presynaptic spike finishes, I_0_ is the triggered maximum EPSC, and I_∞_ is the EPSC at the end of the presynaptic spike. τ is found to be voltage dependent. The drain current, measured from the drain electrode, was shown to be a stretched exponential hump like EPSC when we applied drain voltage on the drain electrode terminal and gate pulse on the gate terminal of a transistor with the source voltage fixed at 0 V. We define the drain voltage as Vd, gate pulse maximum amplitude as Vg, and source as 0.

**Figure 2:**
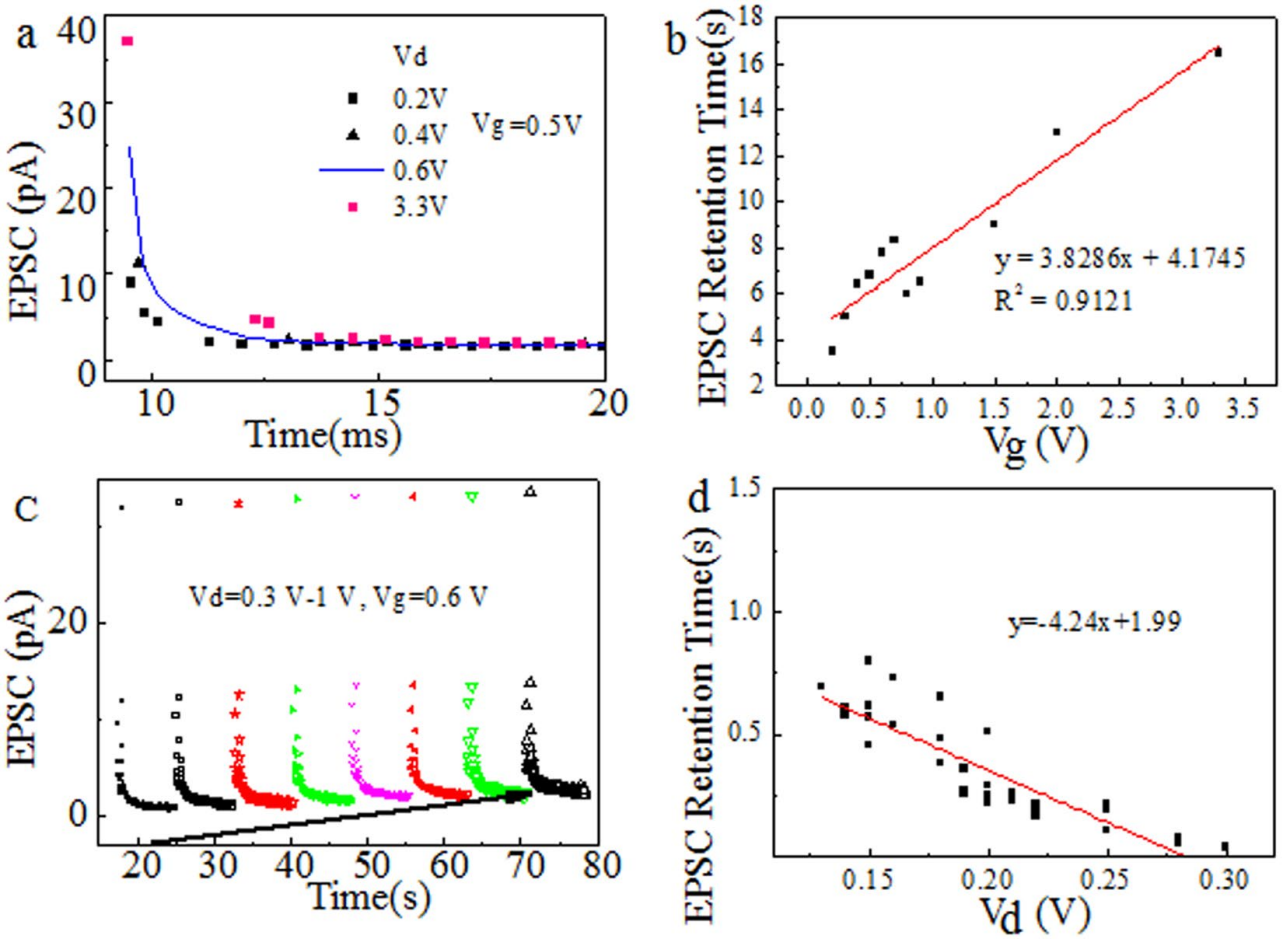
(**a**) Gate voltage dependence of EPSC decay behavior when drain voltage Vd = 0.5 V. (**b**) The gate voltage Vg dependence on retention time. (**c**) Drain voltage dependence of EPSC decay behavior when gate voltage Vg = 0.6 V. (**d**) The drain voltage Vd dependence on retention time.

In order to investigate the impact of voltage on the synaptic transmission behaviors, the voltage on different terminals, i.e., drain electrode and gate electrode, were adjusted and the corresponding transmission behaviors were recorded. The voltage on one terminal was set at a wide voltage value range for CMOS which involve the low working voltage less than 1 V and high working voltage up to 3.3 V, i.e., 0.1~3.3 V. After a pulse applied on the gate terminal, the EPSC current, measured from the drain, was recorded and compared. As shown in Fig. 2a,b, EPSC retention time had uniform and obvious drain voltage amplitude, i.e., V1d, dependence linearly. Therefore, Eq. 1 could be updated as Eq. 2 at a fixed gate voltage.

2 $${\rm{I}}=({{\rm{I}}}_{0}-{{\rm{I}}}_{\infty })\exp [-{(\frac{{\rm{t}}-{{\rm{t}}}_{0}}{{\rm{b}}-{\text{aVd}{\rm{\tau }}}_{0}})}^{{\rm{\beta }}}]+{{\rm{I}}}_{\infty }$$

Similarly, according to Fig. 2c,d, the retention time of EPSC had a gate spike amplitude, i. e. Vg, dependence and Eq. 1 could be updated as Eq. 3 at a fixed drain voltage.

3 $${\rm{I}}=({{\rm{I}}}_{0}-{{\rm{I}}}_{\infty })\exp [-{(\frac{{\rm{t}}-{{\rm{t}}}_{0}}{{\rm{d}}+{\text{cVg}{\rm{\tau }}}_{0}})}^{{\rm{\beta }}}]+{{\rm{I}}}_{\infty }$$

where a, b, c and d are experimental constants. These behaviors are critical as they enable the devices in neuromorphic computers to reach a lot of behaviors such as EPSC with adjustable retention times under the different voltages. Figure 2a,c show that the voltage dependence on drain is less than the gate. It is constant due to the fact that the spike as shown in Fig. 1c could be applied on drain also with a DC gate voltage, but the behaviors are less obvious than those applied on voltage.

**Figure 3:**
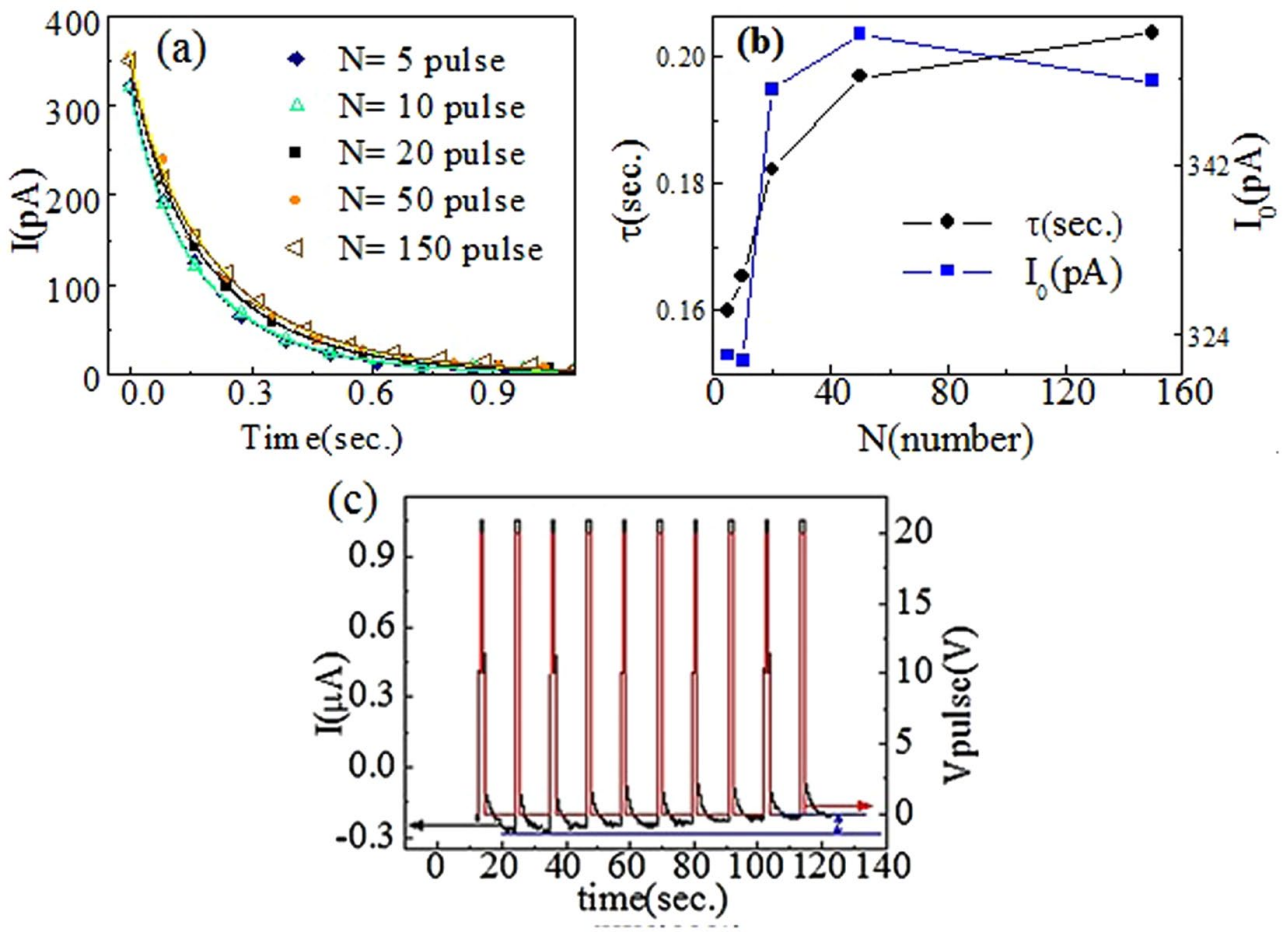
Memory enhancement with repeated stimulation. **(a**) Memory retention data recorded after different numbers of identical stimuli. (**b**) Characteristic relaxation time (τ) obtained through the fitting in panel a and the prefactor (I0) with respect to the number of stimuli (N). (**c**) The corresponding current through the memory device data recorded continuously throughout the test. The spontaneous decay after each pulse (in black lines) and the overall conductor enhancement can be observed. The voltage profile (in red) applied to the memory device consisting of ten 20 V 1.55 s pulses and a constant 0.5 V read voltage.

Memory is an essential function in biological system. In previous biological counterpart, the memory loss is a typical behavior, which can be transformed from short-term memory (STM) into long-term memory (LTM) by the increasing number of stimuli^27^. These behaviors are found in our devices and are expounded in detail. A set of device samples with different pulse number N, i.e., N = 5, 10, 20, 50 and 150 are shown in Fig. 3, suggesting the memory behaviors transfer through repeated stimulation. It is shown in Fig. 3a that the retention loss is correlated with the stimulation pulse numbers. When the pulse number rises, the retention loss increases. This is consistent with the reported memory behaviors for synaptic devices, i.e., the amplitude of which could be adjusted with pulse numbers^21, 27^. The results in Fig. 3b indicate that the retention time increases with increasing the number of stimuli, indicating a decreased forgetting rate. This trend is also consistent to and similar with the memory trends in previous studies, suggesting that amorphous oxide semiconductor transistors would be used as the artificial synaptic devices for investigation and the important role of voltage dependency for the development of nueromorphic computing^21, 27^. Figure 3c shows that upon the application of each stimulation,the off-current increases when the time interval between the stimulation is relatively short. Such behaviors bear resemblance to memory loss in biological system suggests that these devices could to be optimized for memory applications.

The voltage dependence of I_∞_ is also investigated. As shown in Fig. 2, the gate voltage doesn’t change I_∞_ as much as the drain voltage does, whereas the drain voltage have an impact as shown in Fig. 2c. This suggests that Vd could significantly change I_∞_. However, as shown in Fig. 3, I_∞_ also increases when the gate pulse number or the gate voltage stress time, i.e., Vg stress rises. Therefore, Vd, Vg and voltage stress time would impact I_∞_. I_∞_ increases with increasing Vd. Hence the equation could be updated as

4 $${\rm{I}}=[{{\rm{I}}}_{0}-{{\rm{I}}}_{\infty }]\exp [-{(\frac{{\rm{t}}-{{\rm{t}}}_{0}}{(-{\rm{a}}\cdot {\rm{Vd}}+{\rm{b}}\cdot {\rm{Vg}})\cdot {{\rm{\tau }}}_{0}+{\rm{c}}})}^{{\rm{\beta }}}]+{{\rm{I}}}_{\infty }$$

where ${{\rm{I}}}_{\infty }=({\rm{e}}\cdot {\rm{Vd}}+{\rm{f}}\cdot {\rm{Vg}})\cdot {{\rm{I}}}_{\infty 0}$, a, b, c, d, e and f are positive experimental constants.

**Figure 4:**
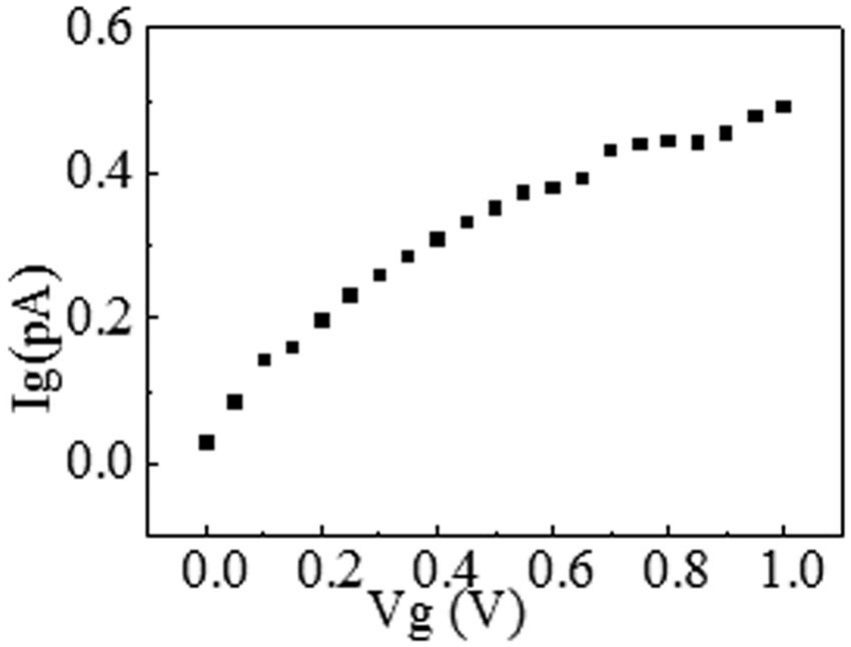
The gate leakage current for Vg = 0~1 V. The small gate leakage current suggests a high quality of the devices, without significant damage by the mobile ions injection and transport.

Besides, as shown in Fig. 3a, the pulse number seems to have less impact on I_∞_ and retention time as compared to previous studies^14, 15^. This illustrates that, under such number of pulses, the amount of mobile charges movements corresponding to the signal is not large enough to cause significant change of retention time. A greater number of pulses or a larger stress is needed in order to obtain an obvious memory effects in our samples. This suggests a high quality of the sample devices without many defects, which may facilitate the carriers and mobile ions to diffuse or transport. This conclusion could be verified with the small gate leakage current which is measured and shown in Fig. 4. In Fig. 4, the gate leakage current is at least 1 order of magnitude smaller than EPSC, which could be excluded. The small gate leakage current suggests a high quality of the devices, without significant damage by the mobile ions injection and transport.

In summary, voltage dependence of retention time is investigated in synaptic transistors. These behaviors suggest that the retentions time are voltage dependent and this voltage dependence could be suitable not only for transistors but also other synaptic devices. The voltage dependence of the synaptic relaxation behaviors under one stimulating pulse could help to obtain a clearer understanding and better controlling of the transition from STM to LTM in synaptic devices.

## Experimental Section

The fabrication process is described subsequently. Using radio-frequency magnetron sputtering method, 30 nm thick IGZO channels were deposited and patterned by lithography on the isolation layer. The channel width and length were about 100 μm and 90–120 μm, respectively. Afterwards, 90 nm thick Ti/Au metal electrodes (i.e., source and drain) were deposited by electron beam evaporation techniques.
