# Author Correction: Realization of tunable artificial synapse and memory based on amorphous oxide semiconductor transistor

**DOI:** 10.1038/s41598-021-91377-y

**Published:** 2021-06-10

**Authors:** Mingzhi Dai, Weiliang Wang, Pengjun Wang, Muhammad Zahir Iqbal, Nasim Annabi, Nasir Amin

**Affiliations:** 1 Ningbo Institute of Materials Technology and Engineering, Chinese Academy of Sciences, Ningbo, 315201 China; 2 Institute of Circuits and Systems, Ningbo University, Ningbo, 315211 China; 3 College of Physics and Electronic Information Engineering, Wenzhou University, Wenzhou, 325035 China; 4 GIK Institute of Engineering Sciences & Technology, Topi, 23640 Khyber, Pakhtunkhwa Pakistan; 5 Department of Chemical Engineering, Northeastern University, Boston, USA; 6 Government College University Faisalabad, Faisalabad, Pakistan

Correction to: *Scientific Reports* 10.1038/s41598-017-04641-5, published online 08 September 2017

The original version of this Article contained an error in Figure 1, where the unit for the y-axis of panel (c) was incorrect. The original Figure 1 and accompanying legend appears below.

**Figure 1:**
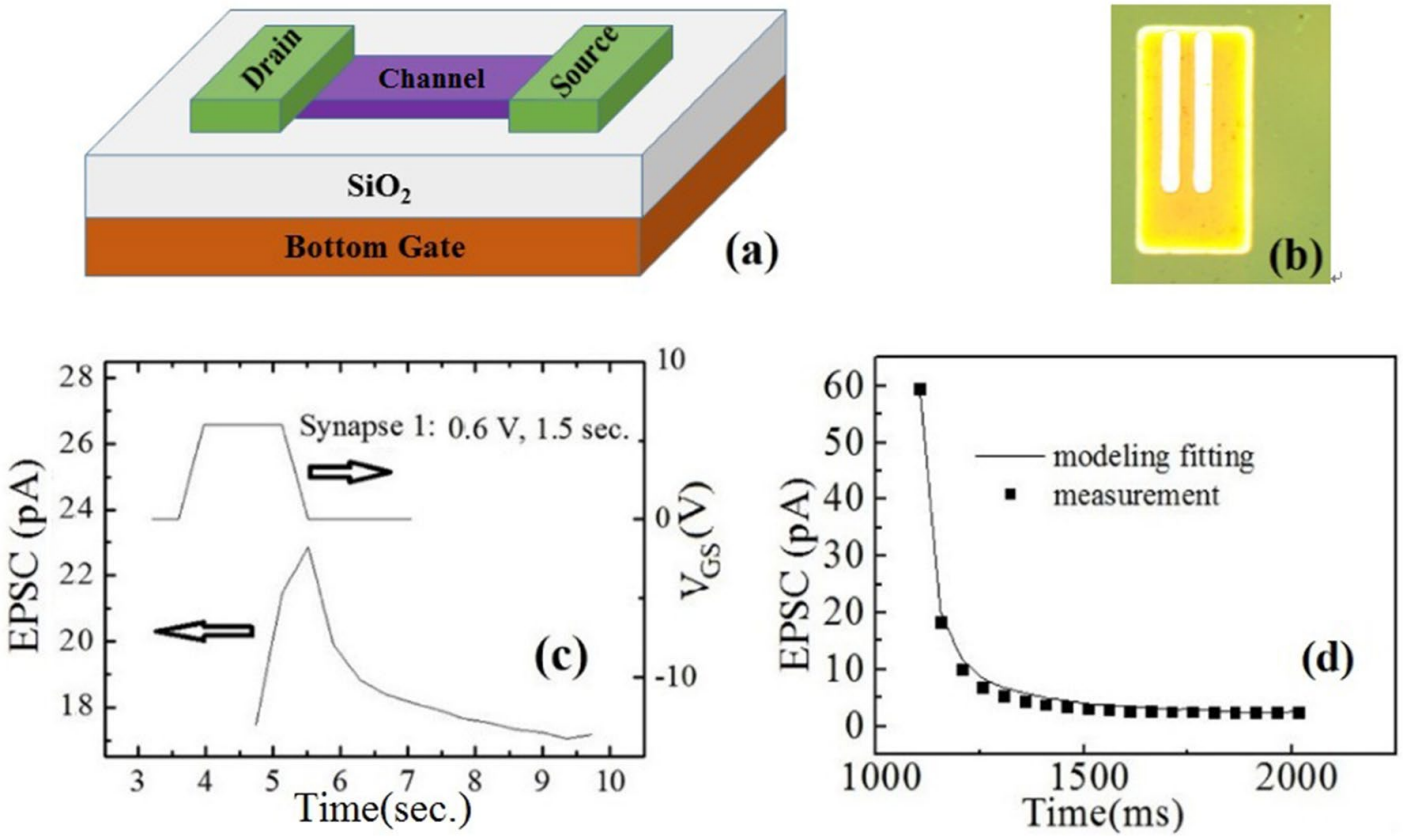
(**a**) The schematic of the 3D structure of the device with bottom gate, source, drain, and semiconductor channel, respectively. The conductive electrodes made of Ni/Au were used. The connection line between the drain and source is the semiconductor channel. (**b**) A representative image form the fabricated device with the bright white metal electrode as long as 800 μm. (**c**) EPSC triggered by presynaptic spike on bottom gate BG. (**d**) EPSC decay behavior with a fitting by stretched exponential function.

In addition, in the Acknowledgements section, the grant numbers for Zhejiang Provincial Natural Science Foundation for Distinguished Young Scholar were incorrect.

“This work was supported by the National Natural Science Foundation of China (Grant Nos 61106090, 61574147, 61474068), Zhejiang Provincial Natural Science Foundation for Distinguished Young Scholar (No. R17F040007), Ningbo Municipal Natural Science Foundation (No. 2014A610011), the Ningbo Natural Science Foundation of China (Grant Nos 2015A610034, 2011A610110, No. 2014B82004, Y10814VA0, 2016A610280), the State Key Basic Research Program of China (2013CB922300), Youth Innovation Promotion Association, Chinese Academy of Sciences and Royal Academy of Engineering, UK. The author appreciates the K.C. Wong Magna Fund in Ningbo University.”

now reads:

“This work was supported by the Zhejiang Provincial Natural Science Foundation for Distinguished Young Scholar (No. LR17F040002), National Natural Science Foundation of China (Grant Nos 61106090, 61574147, 61474068), Ningbo Municipal Natural Science Foundation (No. 2014A610011), the Ningbo Natural Science Foundation of China (Grant Nos 2015A610034, 2011A610110, No. 2014B82004, Y10814VA0, 2016A610280), the State Key Basic Research Program of China (2013CB922300), Youth Innovation Promotion Association, Chinese Academy of Sciences and Royal Academy of Engineering, UK. The author appreciates the K.C. Wong Magna Fund in Ningbo University.”

The original Article has been corrected.

